# Lay social workers implementing a task-sharing approach to managing depression in Vietnam

**DOI:** 10.1186/s13033-021-00478-8

**Published:** 2021-05-29

**Authors:** Leena W. Chau, Jill Murphy, Vu Cong Nguyen, Hayami Lou, Huyen Khanh, Trang Thu, Harry Minas, John O’Neil

**Affiliations:** 1 Faculty of Health Sciences, Centre for Applied Research in Mental Health and Addiction, Simon Fraser University, 515 West Hastings Street, Vancouver, BC V6B 5K3 Canada; 2 Department of Psychiatry, Faculty of Medicine, University of British Columbia, Mood Disorders Centre, 2255 Wesbrook Mall, Vancouver, BC V6T 2A1 Canada; 3 Institute of Population, Health and Development, 14th level, ICON4 Tower, 3 Cau Giay Street, Hanoi, Vietnam; 4 Global and Cultural Mental Health Unit, Centre for Mental Health, Melbourne School of Population and Global Health, 207 Bouverie Street, Carlton, VIC 3053 Australia

**Keywords:** Vietnam, Depression, Task-sharing, Lay social workers

## Abstract

**Background:**

While depression is a leading contributor to burden of disease in Vietnam, there is a critical gap in depression care due to the shortage of mental health specialists and extremely limited mental health services in general health care settings. We have previously reported the effectiveness of a supported self-management (SSM) task-sharing intervention for depression, delivered by social collaborators (lay social workers). The purpose of this study was to identify factors influencing the effectiveness of delivery of SSM by social collaborators and delineate areas for further attention that are relevant for scale-up.

**Methods:**

A hundred and ten (110) key informant interviews were conducted with three stakeholder groups (patients, social collaborators, experts) from eight provinces in Vietnam. Participants were identified through records from a recently completed randomized trial that showed the effectiveness of SSM in community-based settings in Vietnam. Qualitative descriptive methods and thematic analysis were used to examine the interviews. A coding framework and corresponding themes were developed deductively, based on the findings from the randomized trial and the literature, and through inductive analysis, to describe the contextual factors that impacted the social collaborators’ role in successfully implementing the SSM intervention.

**Results:**

Our analysis identified the following benefits of working with social collaborators: (1) *increased awareness of mental health in the family and community*; (2) *reduced stigma*; (3) a *better understanding that depression is treatable*; (4) *increased help-seeking;* and (5) *improved access to care*. There were also significant challenges, including social collaborator characteristics (age, education, pre-existing training and skills) and contextual factors influencing their work (roles and responsibilities, training, compensation, support from government).

**Conclusions:**

Engaging social collaborators in the delivery of SSM in the community can help fill a critical gap in depression care in Vietnam. However, several contextual challenges that are an impediment to increased engagement and sustainable integration into health and social systems need to be resolved through policy change to regulate their practice, define their scope of work, and provide adequate remuneration.

## Background

Depression is a leading contributor to the global burden of disease [1]. While epidemiological data about depression in Vietnam are limited, studies suggest that prevalence is on par with global rates [2], with depression being the second most common mental disorder after alcohol use disorder in Vietnam [3]. Like in many other low- and middle-income countries (LMICs), there is a critical gap [4] in depression care due to the shortage of mental health specialists and extremely limited mental health services in general health care settings [5]. Despite the Government of Vietnam’s progress toward improving the delivery of community-based services through the Community Mental Health Program [6], care for depression is minimally available and services are still concentrated in inaccessible and underfunded tertiary-care facilities [7, 8]. The ongoing COVID-19 pandemic will likely create increased need and demand for mental health services, placing added strain on the existing mental health system. To help address this gap, task-sharing—the expansion of the delivery of health care services by non-specialist providers, including lay health workers, who have received basic training in the context and delivery of an intervention but have received no formal professional certificate or degree [9, 10], has emerged as a viable means to improve access to mental health care in LMICs [11–13].

Despite a large literature on the effectiveness of lay health workers in a number of areas in LMICs, including maternal and reproductive health [14, 15], child health [16], HIV [17], malaria screening and treatment [18], and mental health [19], there is virtually no mention of lay social workers in this literature. However, increased engagement and sustainable integration of lay health workers into national health care systems has been shown to require the development and implementation of policies that regulate and support their work, including incentives affecting motivation, adequate training and supportive supervision, workload, and compensation [13, 20–23] that are consistent with the local economy and environment [24]. Our study demonstrates these same considerations apply to lay social workers.

In Vietnam, the Ministry of Health (MOH) and the Ministry of Labour, Invalids and Social Affairs (MOLISA) are responsible for different but overlapping components of the mental health system. Both have policy mandates to improve community-based depression care. The network of national and provincial psychiatric hospitals and primary care clinics are the responsibility of MOH, while MOLISA is responsible for social workers and overseeing the social protection system that provides rehabilitation and social support services to persons with mental illness [25, 26]. Unique, and relatively new, to Vietnam is a group of lay social workers known as social collaborators who are primarily the responsibility of MOLISA and are mobilized at the community level to provide social support to community members. They are well established now in only a few provinces, but MOLISA is working to expand their contribution to other parts of Vietnam. Social collaborators include retired civil servants, Women’s Union members, ex-village health workers, village Red Cross members, and other local leaders of social groups who volunteer to support other community members experiencing social difficulties. In fact, many perform multiple functions at the community level and may also function as lay health workers, or in other occupational roles. Yet there is substantial variation in their training and experience, scope of work, and remuneration resulting from a lack of regulation and accreditation. For the most part they are unsalaried, although some may receive stipends for carrying out certain tasks [27].

The Mental Health in Adults and Children—Frugal Innovations (MAC-FI) study, funded jointly by Grand Challenges Canada and MOLISA, implemented a community-based mental health intervention as a randomized controlled trial (RCT) that demonstrated improved depression care in Vietnam using task-sharing approaches [27]. MAC-FI included an examination of the contextual factors affecting the integration and sustainability of service delivery by social collaborators in Vietnam’s mental health system. Although there is a growing literature on task-sharing for mental health care using lay health workers in LMICs [19], to the best of our knowledge this is the first research that examines contextual factors influencing integration and sustainability of lay workers in the social services sector for improving mental health care.

The MAC-FI team, composed of Vietnamese (from the Institute of Population, Health and Development [PHAD]), Canadian, and Australian investigators, in collaboration with MOLISA, implemented a decentralized approach to mental health care that included a task-sharing intervention utilizing both the health and social services sectors for training and supervision to deliver a supported self-management (SSM) intervention to adults in community-based settings in Vietnam [5]. SSM, consisting of an *Antidepressant Skills Workbook (ASW)* [28] and supportive coaching, is based on cognitive behavioural therapy principles (e.g., behavioural activation, problem solving, goal setting) and utilizes task-sharing approaches [29], with regular coaching support delivered by social collaborators. Social collaborators were selected by MOLISA to deliver the intervention because they were recognized as trusted community members with experience supporting people in social and emotional difficulty [5]. A provincial-level social worker provided supervision and support to evaluate fidelity. Full details of the SSM intervention are described elsewhere [27]. Results showed that SSM was successful at reducing symptoms of depression when compared with the control group, who received care as usual [27].

The objective of this paper is to identify factors influencing the effectiveness of delivery of an SSM intervention for depression by social collaborators (lay social workers) and to delineate areas for further attention that are relevant for scale-up.

## Methods

Data collection for this paper took place in eight provinces in Vietnam at the conclusion of the MAC-FI study [27]. Semi-structured interviews were conducted with a convenience sample of 110 participants, drawn from the population of patients, social collaborators, and expert stakeholders who participated in the MAC-FI RCT. Forty patients (from 376 in the RCT) and 47 social collaborators (from 336 in the RCT) were recruited from 32 randomly selected communes, two from each participating district. Twenty-three expert stakeholders were recruited from Hanoi, the capital of Vietnam, and from participating provinces.

Patients were identified through records from the MAC-FI study and subsequent monitoring and evaluation of program implementation in the eight provinces. Eligibility criteria included being an adult aged 18 + , having had a score of 7 or over on the self-reporting questionnaire 20-item [30] indicating depression caseness, and having been subsequently referred to the MAC-FI study. Interviews with patients focused on the barriers and drivers to participation and adherence.

The social collaborators were trained as part of the MAC-FI study to deliver SSM and were identified through our ongoing contact with the local health centres where the MAC-FI study occurred. Interviews with social collaborators focused on the challenges and benefits of delivering the intervention at the community level. Patient and social collaborator interviews were conducted by PHAD research staff, with periodic observation by a study investigator.

Purposive sampling was used to recruit expert stakeholders, comprised of primary care and social services managers and policy makers in the health and social services sectors at the provincial and national levels. Interviews with expert stakeholders sought to capture contextual factors influencing the scale-up and implementation of the SSM, including continued delivery by social collaborators. Expert stakeholder interviews were conducted by the study investigators. An interpreter assisted when needed.

### Participant characteristics

Of the 376 patients in the study 84.5% were women and 18.7% were over 55 years (yrs) of age. Of the patients interviewed, 90.0% were women and 37.5% were over 55 yrs of age. Of the 336 social collaborators who worked on the project 81.5% were women and 46.6% were over 55 yrs of age. Of the 47 social collaborators interviewed, 78.7% were women and 48.9% were over 55 yrs of age.

As indicated above, the social collaborator role is relatively new in Vietnam and is only well established in a few provinces. Lay workers in villages often function in multiple roles where they interact with different organizations outside the village to represent their communities’ interests. In our study, some participants were working primarily in the social collaborator role with MOLISA, some in combined roles as social collaborators and village health workers, and some who were recruited and trained to function as social collaborators from other roles in the village, with 35 of the 47 social collaborators interviewed functioning primarily in the social collaborator role. In all cases, social collaborators working on the project were trained in the lay social worker role of providing the SSM intervention to people in the village living with depression. Although social collaborators (and village volunteers generally) rarely have more than basic primary education, in situations where a retired person is functioning in the role, they may have different levels of advanced education and training related to their previous occupation.

Expert stakeholders interviewed included national level and provincial and district MOLISA and MOH staff in senior positions, some of whom were directly involved in the implementation of the project or in the training and supervision of the social collaborators.

Interviews ranged from 30 to 60 min in length and were conducted at a place convenient for the participant. Interview guides were prepared in English and translated to Vietnamese using back-translation for semantic equivalence. All interviews were conducted in Vietnamese, recorded with the permission of participants, and transcribed and translated to English. In instances where the translation was unclear explanations have been included in square brackets.

A national coordinator, based at MOLISA, helped support study recruitment at all levels.

### Analysis

Qualitative descriptive methods and thematic analysis [31, 32] were used to examine the key informant interviews. All interview transcripts were entered into NVivo [33] for coding. A coding framework and corresponding themes were developed deductively, based on the findings from the MAC-FI study and the literature, and through inductive analysis, to identify the contextual barriers to and drivers of the SSM model’s sustainability in its real-life cultural and social context. Team members (HL and LC) independently read, re-read, and coded the translated interview transcripts. Key codes were identified and captured in a coding guide. Through an iterative process involving numerous rounds of discussion and consultation amongst the team members, the codes were identified, categorized, and collapsed into larger themes, which were then discussed and agreed upon by the team. To enhance rigor and trustworthiness, an interrater reliability test was performed and the coding process was documented in detail [34].

All procedures were approved by research ethics boards at Simon Fraser University in Vancouver, Canada [#2016s0604 and 2018s0340] and PHAD in Hanoi, Vietnam [2016/PHAD/MAC-FI-AD-01–01 and 2019/PHAD/IRIS-01]. Funding for the MAC-FI study was provided by Grand Challenges Canada and some of the follow-up interviews were funded by an implementation grant from the Canadian Institutes of Health Research.

## Results

The interviews demonstrated the important role of social collaborators in delivering the intervention. Our analysis identified a number of benefits and challenges in working with social collaborators identified by each participant group (patients [P], social collaborators [SC], expert stakeholders [ES]), which are described below. To ensure confidentiality, we have omitted the participant code.

### The role of social collaborators in the community

Social collaborators spoke of the importance of their existing relationships with patients in their home communities, as described by a social collaborator from Quang Nam:

*That’s why around my neighborhood if anyone had any problems or got sick they would find me for consultation. So many people feel excited when they see me. I do everything with my heart and kindness.* [SC, Quang Nam]

Another social collaborator from Khanh Hoa reported:

*The advantage is that I am living in the area and loved by people so when I come, people welcome me very warmly. […] I also tell people that this is a national project that wants to bring health and wellness to the people in both spirit and body, so please allow me to ask some questions for this project. Please tell me so that I can evaluate and send to the senior level, if there is any problem for you in terms of mental health issues, there will be support. People are very honest and happy.* [SC, Khanh Hoa]

This social collaborator spoke of their unique position in the community and how their existing relationships resulted in people being more responsive, encouraging patients to seek help from their local social collaborators when they were ill, and also suggested that the existing relationships facilitated their ongoing work. This contributed to “the community have[ing] more confidence in social collaborators, strengthening their position in the community”.

The scope of work that social collaborators were responsible for is best described by a social collaborator from Ben Tre:

*My job is to go to meetings, mobilize self-management. […] I said if she [patient] could not go, I was willing to try to relax with her, leading her to walk with a walking stick. Many times, when she lies down at home, she felt depressed, not wanting to contact anyone. […] So I often came to advocate for and motivate her. […] I calm them down, invite them to be happy, suggest we need to still live, then I ask them some questions. I approach the community and ask for support for the people to overcome their mental illness. I support them with everything, from a piece of cake, or rice. […] I approach and confide with such patients. Seeing me, they are very happy because there is no one to talk to.* [SC, Ben Tre]

Participating in the study also led to the development of incentives and team building for the social collaborators. For example, an expert stakeholder from Thanh Hoa province provided an example of a program innovation where a friendly contest was organized for the social collaborators within and across different communities to help them be acquainted with each other and compete to improve the number of people screened:

*Organizing competitions is a custom of the Vietnamese. In these contests, they can directly share their experiences and can talk with each other. These contests are also chances to connect between different residential communities. […] I think that the contest is the effective way of communication and help people know about the project.* [ES, Thanh Hoa]

The contests helped to increase communication and collaboration between social collaborators and helped to increase awareness about the project and mental health and understanding of depression, enhancing trust and improved community connections, supporting their work.

### The impact of social collaborators on community mental health

Social collaborators functioned at several levels to deliver SSM. They provided ongoing supportive coaching, including guidance and encouragement on the various tasks described in the *ASW* through regular visits to the patients’ homes. This engaged process, possible because of the established community involvement of the social collaborators, was considered by participants to have contributed to the effectiveness of the intervention. Social collaborators being located at the community level was seen as being conducive to frequent patient visits, which ranged from several times each week, especially in the beginning, to once a month, and helped to build “good relationship[s]”. One social collaborator from Quang Nam indicated that “sometimes she [her patient] calls me and wants to hang out, sing karaoke with me.” Social collaborators were unanimous in indicating that they dedicated a lot of their time, especially at the beginning of the intervention, to visiting patients. During their visits, they discussed the *ASW*, “encourage[ing] [patients] to finish the book and learn positive principles,” providing suggestions for hobbies to keep patients engaged, such as karaoke or swimming, or simply “talk[ed] to them”.

Social collaborators were described as having a particularly important impact on: (1) *increased awareness of mental health in the family and community*, (2) *reduced stigma*, and (3) a *better understanding that depression is treatable*.

As an expert stakeholder in Hanoi explained:

*After knowing their problems, they received materials from social collaborators and they know how to take care [of their] depression. […] This project helps to raise the awareness of people about depression. […] After being counselled by social collaborators, their patients’ families don’t have any stigma with depressed people. Before that, they don’t know and often have superstition, [believing] that the reason of the problem is ghosts and they look for sorcerers. And now they know what depression is and coordinate with social collaborators.* [ES, Hanoi]

While some patients reported that they and their families had previously attributed mental illness to cultural beliefs about sorcery, the social collaborators helped them to increase their awareness of mental health, supporting “patients’ family members to understand and know how to better care for the patients,” and that treatment was possible. This awareness and understanding helped to reduce stigma about mental illness amongst patients themselves, their families who serve a variety of important roles in the patients’ lives, and in the wider community.

An expert stakeholder in Thanh Hoa province commented on reduced stigma in the community:

*The patients who were exposed to the program and received support from our staff, they have changed significantly and the stigma in the community is also reduced markedly. Previously, some patients were shunned because of several reasons. Firstly, some people think that they might be infected. Secondly, some patients cannot control their behaviors and sometimes can have quarrels. After our project, people have more sharings [shared understanding of mental health].* [ES, Thanh Hoa]

A patient from Da Nang emphasized they “want the program to be expanded” as they “think it’s useful for many people” by highlighting the beneficial impact that increased awareness about mental health and the options to treat and manage depression can have for an individual:

*When someone has conditions that they will be depressed, if they are given this information they will understand, depression is milder. In the past, I used to sit in the corner, there was no contact with anyone, I thought I had cancer and was going to die.* [P, Da Nang]

Social collaborators were recognized for contributing to: (4) *increased help-seeking* and (5) *improved access to care*.

There was *increased help-seeking* amongst the patients arising from the increased mental health awareness in the communities. An expert stakeholder in Khanh Hoa province stated “this year, there are many cases of depression in the area. Last year we also went but we could not find any cases, did not cover all the communes, but this year they discovered a lot of people participating in the program.” An expert stakeholder from Hanoi provided a reason for the increased number of patients with depression seen in the program:

*Depression patients are instructed by our social collaborators on how to overcome their situation. They now know the symptoms of depression in the early stages, and then they will go to commune health centers to seek advice, while before this model [SSM], they don’t know what depression is. Depress[ed] people feel more confident and they know their problems.* [ES, Hanoi]

SSM delivered by social collaborators was described as *improving access to care*, particularly for individuals in rural communities. An expert stakeholder from Thanh Hoa stated that “I just think that this model targets those who are in the early stage of depression and if we can’t control depression in the early stage, patients can be much more severe in the level of depression.” Social collaborators helped to target depression in the early stages by working closely with community members who they knew to be experiencing social and emotional difficulties, and they were able to devote a large amount of time to visiting their patients in their homes. As a patient from Thanh Hoa reported, “In general, since this program began, there are some collaborators who help me every week. I felt better.”

### Challenges for program scalability with social collaborators

Although the MAC-FI trial [5] demonstrated that a community-based mental health intervention supported by social collaborators results in improved mental health, and this study has identified which aspects of the social collaborators’ role are regarded as having contributed to that outcome, we also identified factors at the system level that inhibit the full implementation of this intervention.

#### Age

Age was a complicating factor for social collaborator effectiveness. The age of social collaborators ranged from 37–70, with 46.6% over the age of 55.

While older retirees were well known and respected in communities, which facilitated their ability to gain patients’ trust, older age was also associated with difficulties in acquiring the new knowledge required for the intervention. As an expert stakeholder from Quang Ninh indicated, the “collaborators are mainly retirees” who are able to “take advantage of the part-time job”, with payment in the form of task-specific stipends.

Some social collaborators and expert stakeholders acknowledged that older collaborators’ “acquisition [of new skills] is limited because of their age”. For example, an expert stakeholder from Thanh Hoa commented that, while they recognize qualified social collaborators without special health training are able to provide effective service, they purposely tried to recruit younger social collaborators to facilitate faster learning and comprehension. This person suggested it was more difficult for social collaborators of an advanced age to learn new information:

*Secondly, as for the selection of social collaborators and communes, we should choose the collaborators who are in the ages that are easy to learn something new. […] If they are capable and are qualified, they can participate really well. […] In our project, there are medical personnel called village health workers, despite advanced ages, they are still involved but their performances were not good enough. Therefore, when our project continues, the recruitment of collaborators should select those who are qualified and in a young age to meet the requirements of the program.* [ES, Thanh Hoa]

The challenges were also reported by social collaborators themselves. For example, a social collaborator from Long An stated:

*I think young people like you [referring to the interviewer] are very talented. Old person like me cannot be talented like you. In fact, people said that they would like to see the old doctor, because they are full of experience, but I think there is no experience because the things they have learnt in the past is different from now. Now when I went to big hospital like Thong Nhat Hospital, it is full of talented young doctors, I found that the youth is talented.* [SC, Long An]

Thus, while age and the related experience was definitely an advantage in encouraging trust with communities, our study also suggests that scaling up the intervention might require the development of training programs that are adapted for older learners. Conversely, while young people might learn more quickly, they might not connect as well with patients and may need more training and support to establish trusting relationships with people suffering from depression in the community.

#### Education and training

The formal education that social collaborators had previously received was varied. One social collaborator from Quang Nam expressed concern regarding their level of education, “my education level is not high. I talk not well which can make many patients misunderstand.” Some social collaborators reported that they had difficulty communicating with patients and perceived this to be due to their limited education level. An expert stakeholder from Quang Ninh reported that education in “the remote areas sometimes ends only in the ninth grade.” Another expert stakeholder from Thanh Hoa indicated:

*As for the difficulties, staff working in the villages have different levels of education and different age. For example, the number of village health workers finishing college is quite low. […] Because of the different levels of education, the screening of patients is different among different staff.* [ES, Thanh Hoa]

The absence of formal education among the social collaborators was seen by some as contributing to differences in patient trust. One expert stakeholder from Quang Nam said, “there are a lot [of] collaborators [with] whom patients didn’t want to cooperate. Quang Nam still lacks a professional collaborators system in the community. There are a lot of issues in this project because we just could mobilize village staff.”

Similarly, pre-existing training and skills of the social collaborators, while varied, was also limited, which contributed to challenges in delivering the intervention. This is not surprising considering that many were retirees with no specialized training prior to the study, with a number of social collaborators indicating their previous responsibilities as “just farming,” “housewife,” “work[ing] in the countryside and sell[ing] rice”, “garden[ing]”, and being “a soldier”. This is compounded with “limited” and “short-term training programs” that could result in barriers to implementation. Similar to the challenges associated with age, the introduction of a professional development program for social collaborators, geared to their level of formal education, would further support the scalability of this intervention.

### Contextual factors influencing scalability of working with social collaborators

There were additional challenges highlighted by participants arising from a lack of policy and regulation for social collaborators around their roles and responsibilities, training and supervision received, and remuneration.

#### Roles and responsibilities

Social collaborators had to juggle numerous work and personal responsibilities, such as harvesting. An expert stakeholder from Hanoi stated that the main barrier they saw to the project’s scalability was the workload of the social collaborators: “They are working for the project but when it comes to the time for harvesting, they don’t have enough time to go to the house to ask and screen patients,” which leads to high turnover. An expert stakeholder from Quang Ninh similarly reported, “the capacity of a collaborator is limited because they have too much work”. This was emphasized by a social collaborator from Quang Nam: “I just want to talk about the collaborators, they were enthusiastic, but they did not have time.”

#### Training

Training available to social collaborators was variable across provinces. One expert stakeholder from Thanh Hoa indicated, “in addition to the training programs from the Institute of Population, Health and Development, we also have many other training programs related to this problem such as training programs from DOH [Department of Health]. In our center, we also have training programs for our staff.” This was not reflected consistently across the province or in other provinces. One expert stakeholder from Thanh Hoa pointed out, “there are some limits in their training programs and their implementation of the screening questionnaire” and “they just received short-term training programs”.

DOH officials were also concerned about the social collaborators’ lack of “knowledge about medicine” that “hindered their ability to deliver the intervention and their patients’ trust in them”. This resulted in patients at times “discriminat[ing] [against] the collaborators and closing their doors” as they were not receptive to their services, with a number indicating they instead preferred “treatments with medicine” or “the hospital”, highlighting the point that some patients prefer a provider they perceive as an expert, which fits with a more medical model of mental health care.

In our study, province-level social workers provided most of the supervision for the social collaborators. However, at a country level, supervision varied across provinces and communes as the capacity among the social workers also differed, emphasizing the need for enhanced training for both social collaborators and the social workers supporting them. As suggested by an expert stakeholder from Thanh Hoa:

*Additionally, in order to have effectiveness for patients, it could be necessary to have training programs for provincial supervisors and collaborators, which are not one- or two-days training sessions as we did. The training programs should be long-term training because [increasing knowledge][…] cannot be [achieved] in one or two days.* [ES, Thanh Hoa]

#### Payment

Social collaborators received a small stipend from the project of approximately 30,000 VND [equivalent to roughly CAD$1.70] per patient. Although the amount of the stipend was determined by MOLISA staff as consistent with other stipends provided to social collaborators and village health workers, respondents indicated this was “not commensurate with their effort” given their frequent patient visits. Despite the fact that social collaborators “work because of the passion”, a lack of, or insufficient, payment poses a particular problem considering that social collaborators often “have many things to do” and would need to prioritize tasks that provide a living.

An expert stakeholder from Thanh Hoa said, “Because the funding spent to support collaborators is still limited, they are not very enthusiastic about our activities.” The lack of core salary funding from government, combined with a lack of coverage by health insurance programs for mental health services, may not provide sufficient motivation for this provider group. This will be a challenge for sustainability, as a model dependent on a system of volunteers is unlikely to be sustainable [35]. As an expert stakeholder from Khanh Hoa stated bluntly, “They are very enthusiastic now, but if they have money they will be more enthusiastic.”

#### Support from government

As noted by an expert stakeholder from Hanoi: “The second weakness of this project is the coordination between multiple sectors such as health sector, education and mass organizations because sometimes our social collaborators cannot take the responsibility to do all the things and they need to transfer their jobs to other sectors.” Improved coordination between sectors would provide clarity on their role.

The overarching challenge to working with social collaborators, not just to deliver SSM but more broadly, is the fact that “there is no policy about social work,” as reported by an expert stakeholder from Quang Nam. Without a social work policy that clearly defines their roles and responsibilities, training and supervision available, and payment, there will be challenges to their full engagement and integration into the health system. The social collaborators’ broad scope of work was undefined and unregulated, which can impact quality of care and potentially lead to disempowerment. These factors may have contributed to the high turnover rate of social collaborators that was observed.

## Discussion and recommendations

Social collaborators can play an integral role in task-sharing models of delivery of effective mental health care and social support to adult populations in community-based settings in Vietnam. Studies have reported that trained non-specialist providers in primary care clinics helped to reduce depressive symptoms in adults [36] and improve outcomes for depression, post-traumatic stress disorder, and alcohol use disorder [37] in LMICs. The MAC-FI study demonstrated that, despite the challenges identified, lay social workers (social collaborators) can provide effective social support to improve mental health care via SSM to those with mild to moderate depression, with training and supervision from a social worker [27].

Social collaborators helped to overcome the challenge of low help-seeking for depression [38], fostering trust and engagement, highlighting the importance of improved communication and advocacy in the communities to increase mental health awareness. Their dedication to visiting patients on a regular basis resulted in improved access to care. However, successful implementation, scale-up, and sustainability of the SSM model delivered by social collaborators will require a number of key contextual factors to be addressed. These include training and supportive supervision, regulation defining scope of work and remuneration, and policy support from government.

### Training and supervision

There is a need to engage the social services sector to provide ongoing training for social collaborators to deliver the intervention and for more experienced and better trained social workers to provide training and supervision [13]. While the social collaborators who participated in our study received on-the-job training in areas specific to the implementation of SSM, enhanced training and supportive education in the general areas of health promotion and disease prevention may help increase social collaborator satisfaction, and reduce burnout and turnover [39]. Enhanced and ongoing “booster” training to improve knowledge and build practical skills for delivering community-based mental health care will likely provide future employment opportunities for social collaborators, indirectly strengthening their social and family status.

Supervision is a crucial aspect to supporting social collaborators in their work and keeping them motivated and engaged [13, 40]. A social support model, wherein the usual providers such as social workers provide ongoing supervision for social collaborators, was shown to be necessary [41]. A sustained task-sharing model requires better trained social collaborators, with consistent support and supervision by social workers to provide oversight for quality assurance.

Although not addressed in our studies, mobile health (mHealth), an advancing field that has been shown to improve healthcare delivery in resource-limited settings [42, 43], has been suggested as a viable method for delivering the required training and supervision. Supports could include online courses, training through mobile screening applications, or online booster coaching to further improve the quality of services while reducing costs of training [44, 45]. There are, however, some key considerations to address, including IT literacy among lay health workers, cellular network coverage and electricity access, and phone credit financing [45].

### Acceptability of social collaborators by patients and the community

The three stakeholder groups reported numerous community benefits of social collaborators delivering the SSM intervention, including increased awareness of mental health in the family and community and improved access to care. However, a number of stakeholders reported some patients preferring a provider whom they perceived as a medical expert over a social collaborator, which is a reflection of the preference of some individuals for a more medical model. This may be a barrier to some patients seeking help from a social collaborator but results showed patients predominantly appreciated the support they received from social collaborators and were willing to seek help and learn skills from them.

### Regulation defining scope of work and remuneration

Consistent with the literature on lay health workers emphasizing the added burden of delivering additional health services onto their existing workload [39], social collaborators are inadequately compensated and overburdened with multiple responsibilities. Regulation, which will require increased coordination and collaboration between MOH and MOLISA, is needed to improve policies on scope of work, training, and remuneration. Other studies have argued that remuneration is a crucial aspect to scale-up and sustainability as task-sharing cannot be sustained only by a system of volunteers who do not receive fair compensation supporting their livelihood [35]. A key motivation for lay health workers including social collaborators is alleviation from poverty [23], which can result in the possible exploitation of the poor for frequently unpaid, almost invariably under-paid and at times specialized work that should be funded by government. Regulation is also required to fully integrate social collaborators into health and social systems that overlap in provision of mental health care. Working with government to implement policy change will be key to improving community-based depression care in Vietnam, which requires flexibility and an understanding of the realities of changing contexts, including the economic and health systems consequences of the ongoing COVID-19 pandemic. Findings from MAC-FI can provide strategic opportunities for engagement in policy development for enhanced mental health services in Vietnam.

### Support from government

To facilitate the task-sharing process and to support improved overall mental health service delivery in Vietnam, referral processes between the social (MOLISA) and health (MOH) sectors need to improve to enable timely and appropriate care for individuals with more severe depression. Inter-ministerial collaboration remains a challenge in Vietnam, as in many other countries, and will be necessary to promote effective referral pathways and continuity of care [21].

While a focus on sustainability is a primary recommendation in the context of task-sharing [9], it is challenging in practice [46]. There is a need for better understanding of the factors that influence scale-up and sustainability of task-sharing in mental health [47].

## Strengths, limitations and future directions

Although this study focused specifically on task-sharing to improve depression services, the barriers and drivers mentioned could apply, as indicated in the literature, to other health issues in Vietnam and other LMICs. The descriptive and thematic analyses provide an insight into the work of social collaborators via task-sharing in the context of delivering a SSM intervention to improve community-based depression services in Vietnam. The research team is currently examining the scale-up process in real time and in the real-world context in an implementation study.

Social collaborators were chosen based on their established relationships and leadership within the community, resulting in a convenience sample. Thus, we were not able to control for any characteristics of the social collaborators, as mentioned in the results section, resulting in substantial variability in their age, education, and pre-existing training and skills. In addition, because they were essentially volunteers who were paid a small stipend, we were not able to systemize their involvement and delivery of the intervention.

Although this study did not include a cost–benefit analysis, the team plans to conduct one for scale-up and sustainability of SSM delivered by social collaborators, including training of social workers to provide supervision and quality assurance, supporting staff, and turnover. Additionally, amidst current funding constraints, the team plans to expand the reach of SSM in rural communities by conducting a pilot examining the feasibility and cost-effectiveness of training social collaborators using smartphones and delivering SSM virtually via a mobile app.

## Conclusions

Engagement of social collaborators (lay social workers) to deliver the SSM intervention has important implications for improving availability of, and access to, depression care in Vietnam. Despite the challenges identified here, the SSM intervention demonstrated significant improvement for patients [27]. This model, involving social collaborators, can help fill a critical gap in care for mild to moderate depression in Vietnam and other low-resource contexts. By offering accessible and low-cost depression care in rural communities where services are often unavailable, SSM, delivered by social collaborators, is reported to have helped to increase mental health awareness and reduce social marginalization and stigma in patients and their communities in Vietnam.

However, there are several key systems-level contextual challenges to increased participation by, and sustainable integration of, social collaborators, which require the development and implementation of context-specific policies [24] that regulate their work, including a manageable workload within clearly defined roles and responsibilities, training and supervision received, and appropriate remuneration. The social collaborator role is new in Vietnam and will need to be expanded and supported if a social support approach to community mental health embedded more in a social work rather than medical model is to further develop. This model has substanstial potential for mental health and psychosocial support in low resources settings, and futher analysis and studies to determine if this model can be translated into other LMICs is needed.

Similarly, the World Health Organization has recently called for a further examination of cross-cutting contextual factors including management and supervision, training, accreditation and regulation, and motivation and remuneration that support global scale-up and sustainability of lay health workers [23, 48]. The involvement of both the health (MOH) and social services (MOLISA) sectors was crucial to the success of MAC-FI, helping to contribute to more coordinated, rather than siloed, approaches to mental health care provision. Sustained engagement and change involving both sectors to develop and implement policies to regulate social collaborators, define their scope of work, provide adequate remuneration, and fully integrate them into health and social systems will contribute to overall health systems strengthening.

## Data Availability

The datasets generated and/or analysed during the current study are being stored in Simon Fraser University’s RADAR research data repository. Access to the datasets is available to members of the study team.

## Abbreviations

LMICs: Low and Middle-Income Countries
MOH: Ministry of Health
MOLISA: Ministry of Labour, Invalids and Social Affairs
MAC-FI: Mental Health in Adults and Children-Frugal Innovations
RCT: Randomized control trial
PHAD: Institute of Population, Health and Development
SSM: Supported Self-Management
ASW: Antidepressant Skills Workbook
P: Patients
SC: Social collaborators
ES: Expert stakeholders
DOH: Department of Health
